# Sexuality and fertility desire in a large cohort of individuals with 46, XY differences in sex development

**DOI:** 10.1016/j.clinsp.2023.100185

**Published:** 2023-03-23

**Authors:** Rafael Loch Batista, Marlene Inácio, Vinicius Nahime Brito, Maria Helena Palma Sircili, Min Jeong Bag, Nathália Lisboa Gomes, Elaine Maria Frade Costa, Sorahia Domenice, Berenice Bilharinho Mendonca

**Affiliations:** Developmental Endocrinology Unit, Hormone and Molecular Genetics Laboratory (LIM/42), Endocrinology Division, Internal Medicine Department, Faculdade de Medicina da Universidade de São Paulo, São Paulo, SP, Brazil

**Keywords:** Sexual life, Differences of sex development, Human sexuality, Sexual orientation, Gender change

## Abstract

**Objective:**

To analyze aspects of sexual life and fertility desire among 46, XY DSD people, including those who changed their gender.

**Methods:**

It is a cross-sectional study including 127 adults (> 16 years of age) with 46, XY DSD (83 females; 44 males) from a Single Brazilian Tertiary-Care Medical Center.

**Results:**

Sexual fantasies and masturbation were more frequent in 46, XY DSD males, whereas orgasm and sexual life satisfaction were similar in both genders. More 46, XY DSD men than women had a long-term romantic relationship. 46, XY DSD women with prenatal androgen exposure reported more fear of being romantically rejected. External genitalia appearance at birth did not impact the sexuality of 46, XY DSD women after surgical genital treatment had been completed. Overall, the sexual life was similar between 46, XY men assigned as males and those who changed to the male gender. Regarding sexual orientation, most self-reported as heterosexual (91% and 92% of women and men, respectively). The desire for fertility had a similar prevalence in both genders, but more women than men considered infertility a barrier to a long-term romantic relationship. Twelve individuals (7 males) had children; 10 out of 12 have adopted children.

**Conclusion:**

Fertility desire was shared among 46, XY DSD people, regardless of gender. Prenatal androgen exposure reduced the desire for motherhood in 46, XY women. 46, XY DSD people who changed from female to male gender presented similar sexual parameters as those assigned as males. Among females, virilized genitalia at birth did not affect sexuality once the surgical treatment is completed.

## Highlights

• Prenatal androgen exposure reduced the desire for motherhood in 46, XY women.
• 46, XY DSD people who changed from female to male gender presented similar sexual parameters as those assigned as males.
• Among females, virilized genitalia at birth did not affect sexuality once the surgical treatment is completed.
• Fertility desire is shared among 46, XY DSD people, regardless of gender.

## Introduction

One of the critical points in the care of patients with DSD is regarding sexuality,^1^, ^2^ which can be negatively impacted by many factors, including external genitalia appearance, prenatal steroids exposure, the incongruence between gender identity and sex assignment, negative body image, traumas, social stigma, poor familial environment, and previous genital surgery.^3^, ^4^ In addition, all these factors can potentially promote psychological issues and sexual dissatisfaction in these individuals.^3^, ^5^

Additionally, gender change occurs across 46, XY DSD conditions, from female to male and male to female.^6^, ^7^ As most 46, XY DSD individuals were assigned as females in the past, female-to-male change is much more common than the opposite in this population.^7^, ^8^ This change occurs especially among 46, XY DSD conditions associated with prenatal androgen exposure, such as 5α-reductase type 2 deficiency and 17β-hydroxysteroid dehydrogenase type 3 deficiency.^7^, ^9^, ^10^ However, data about the sexuality of 46, XY DSD individuals who changed their gender is lacking in the literature.

In this study, the authors aimed to analyze the sexual aspects (sexual life behavior and sexual orientation) and fertility desire of 46, XY DSD individuals assigned to both genders, including those who changed from female to the male gender. In addition, the authors evaluated the influence of prenatal androgen exposure based on 46, XY DSD diagnosis and virilized external genitalia at birth on the sexuality of female 46, XY DSD patients were analyzed.

## Patients and methods

One-hundred and fifty-five followed by a multidisciplinary team at a single Brazilian tertiary-care medical center (Hospital das Clínicas, Medical School, Universidade de São Paulo, Brazil) were invited to participate in this study. A total of 127 agreed to participate in this study. All of them had 46, XY karyotypes. Only adult individuals (more than 16 years of age) with 46, XY DSD due to seven 46, XY DSD etiologies were enrolled, most of them with molecular diagnosis (Table 1). The mean chronological age of male and female DSD patients was 37 and 35 years, respectively. The authors performed the sequencing of 46, XY DSD candidate genes in the whole cohort. One hundred and seven (84%) individuals were assigned females at birth, whereas 20 (16%) were assigned, males. All patients who had genital surgery indicated had completed surgery at the time of inclusion in this study. The Local Ethics Committee approved the study, and all patients agreed to participate and signed the written consent term.

**Table 1:** Frequency of each 46, XY DSD etiology according to sex assignment.

	Sex assignment		Gender in adulthood		
DSD etiology	Female	Male	Female	Male	Total
5α reductase 2 deficiency	29	1	13	17	30
17β HSD3 deficiency	15	‒	10	5	15
17α/17,20 liase deficiency	7	‒	7	‒	7
Leydig cell hypoplasia	7	‒	7	‒	7
Complete gonadal dysgenesis	7	‒	7	‒	7
Partial gonadal dysgenesis	12	7	9	10	19
CAIS	18	‒	18	‒	18
PAIS	12	12	12	12	24
Total	107	20	83	44	127

A self-developed questionnaire on sexual life aspects was applied by the same investigator (Inacio M), a clinical psychologist with expertise in DSD. To develop the questionnaires that were used in this study, the authors collected similar questions from previously published papers regarding the sexuality of 46, XY DSD people from 1974 to 2022.

For comparison of the sexual life parameters, the individuals were divided into three groups according to the following criteria: group A gender in adulthood: female (n = 107) vs. male (n = 20), to compare differences regarding sex assigned; group B external genitalia appearance at birth in female 46, XY DSD individuals: typical female genitalia (n = 60) vs. atypical external genitalia (n = 35), to analyze the impact of atypical external genitalia on female sexuality; and group C male gender in adulthood: 46, XY DSD individuals who were assigned male at birth (n = 20) vs. 46, XY DSD individuals who changed from female to male gender (n = 24), to evaluate the impact of gender change on their sexuality.

The degree of external genitalia undervirilization was assessed by Synnecker score,^11^ which works on a Likert scale ranging from 1 (typically male external genitalia) to 5 (typically female external genitalia).

The chronological age at starting sexual life was self-reported. The sexual questionnaire contained the following questions with a dichotomous response (yes/no): Do you have an active sex life? Are you satisfied with your sex life? Have you been in a long-term romantic relationship? Do you have sexual fantasies? Do you practice masturbation? Do you have an orgasm? Do you be afraid of being sexually rejected? Do you feel ashamed of your body?

The frequency of sexual activity was measured as follow: What is the monthly frequency of sexual act? (1 – once; 2 – twice; 3 ‒ three times; 4 ‒ four times; 5 ‒ more than four times).

Sexual orientation was assessed by both self-reported sexual orientation and sexual behavior. For self-reported sexual orientation, the patients answered the question: What is your sexual orientation? The answer was composed of four categorical options (heterosexual, homosexual, bisexual, or asexual). Sexual behavior was assessed through the question: Which gender do you practice sex? The answer was based on a Likert scale (to female gender individuals: 1 = exclusively women; 2 = more women than men; 3 = equal men and women; 4 = more men than women; 5 = exclusively men; and the opposite for male gender individuals: 1 = exclusively men to 5 = exclusively women).

Fertility issues were accessed based on the following questions after previous information regarding fertility desire and fertility potential: Do you have children? If yes, the pregnancy was: 1) Natural; 2) Induced by assisted reproductive techniques, or 3) Adopted child. If not, do you have a fertility desire? If yes, would you like to have biological children? In case of infertility: would you like to adopt a child? Are you afraid to be rejected due to infertility? How important is fertility for you (Likert scale from 1‒ it is not important to 5 – it is very important). Do you think infertility is a problem for a long-term relationship (Likert scale from 1 ‒ it is not important to 5 – it is very important).

Finally, to assess the impact of prenatal androgen exposure on sexuality, the authors divided the female patients into two subgroups, as follows: without prenatal androgen exposure (n = 41: 24 Complete Androgen Insensitivity Syndrome (CAIS), seven complete gonadal dysgeneses, 7 Leydig cell hypoplasia, 4 with 17α-hydroxylase deficiency with typical female external genitalia) and with usual male prenatal androgen exposure (n = 14; all of them with 5 alpha-reductase type 2 deficiency diagnosis).

### Statistical analysis

The Shapiro-Wilk test was used to test the normal distribution of continuous variables. Descriptive statistics are presented as mean and standard deviations. Associations between categorical variables were analyzed using Chi-Square tests and contingency tables (2 × 2).^12^ All analyses were performed using the SPSS 27.0 statistical analysis package (SPSS, Chicago, IL). Statistical significance was set at p < 0,05.

## Results

The frequency of each 46, XY DSD diagnosis is summarized in Table 1. As far as those individuals who did not agree to participate in this study, the most frequent diagnosis was complete gonadal dysgenesis (n = 7), followed by complete androgen insensitivity (n = 6), partial androgen insensitivity (n = 4), 3β-hydroxysteroid dehydrogenase type 2 deficiency (n = 3), 17α-hydroxylase deficiency (n = 2), 5 α-reductase type 2 deficiency (n = 3), 17β-hydroxysteroid dehydrogenase type 3 deficiency (n = 2), and partial gonadal dysgenesis (n = 1). In 22 cases (78%) they were not available to attend the hospital in person at the time of this study include. One patient (a female with 5 α-reductase type 2 deficiency) refused to participate because she is a nun from a catholic church. Other five individuals because they had not started their sex life at the time of this study (1 female patient with complete gonadal dysgenesis, 1 female patient with partial androgen insensitivity, 1 female patient with 17β-hydroxysteroid dehydrogenase type 3 deficiency, 1 female patient with complete androgen insensitivity, and 1 male patient with partial gonadal dysgenesis). Regarding female patients who had undergone feminizing genital surgery, the average age of surgery was 13.85 years (±9.71). The average number of genital surgical procedures was 1.31 (minimum 1, maximum 3, mode 1). Surgical procedures were performed during childhood in 14 patients (40%), at puberty in 6 patients (11‒16 years of age, 17.1%), and adulthood in 15 patients (42.9%). The clitoral reduction was performed in 31 (88.6%), vaginoplasty in 3 (8%), and labioplasty in 10 (28.6%). Vaginal dilation was necessary for 13 patients (37.1%) before starting sexual activity. The 46, XY DSD diagnoses of the female individuals who had genital surgery were: partial androgen insensitivity (n = 11, 31.4%), partial gonadal dysgenesis (n = 9; 25.7%), 17α-hydroxylase deficiency (n = 2; 6%), 5 α-reductase type 2 deficiency (n = 6; 17.1%), 17β-hydroxysteroid dehydrogenase type 3 deficiency (n = 7; 20%).

### Sexual life in 46, XY DSD

#### Gender in adulthood (male vs. female)

The mean chronological age of the onset of sexual life was 22 (± 2) and 21 (± 1.5) years in the female (n = 107) and male gender (n = 20), respectively (Table 2).^13^ There was a statistical difference in the frequency of sexual fantasies (40% in females vs. 85% in males; p = 0.004), masturbation practice (39% in females and 75% in males; p = 0.01), and prevalence of long-term sexual partner (48% in females vs. 70% in males; p = 0.003), between both genders. However, sexual frequency, satisfaction with sexual life, and orgasm were similar in both genders (Table 2).

**Table 2:** Sexual Life Parameters in 46, XY DSD Individuals according to gender at adulthood, external genitalia appearance at birth, gender change, and prenatal androgen exposure.

	Gender			External genitalia appearance at birth			Prenatal androgen exposure			Gender Change		
Sexual life parameters	Female	Male		Atypical	Typically female		No	Yes		Assigned as male	Change to male	
n	107	20	p	35	60	p	35	14	p	20	24	p
Body shame (%)	30	40	0.95	31	33	0.75	31	36	0.44	25	37	0.43
Afraid of rejection (%)	16	25	0.8	17	20	0.24	17	43	0.001	25	8	0.07
Long-term romantic partner (%)	48	70	0.003	46	49	0.75	46	36	0.67	40	62	0.13
Masturbation (%)	39	75	0.01	40	55	0.52	40	43	0.21	65	67	0.9
Sexual fantasies (%)	40	85	0.004	51	45	0.21	51	43	0.51	70	54	0.09
Orgasm (%)	66	80	0.6	83	90	0.93	83	71	0.62	85	91	0.8
Active sexual life (%)	65	85	0.13	71	80	0.75	71	43	0.35	75	96	0.04
Sex life satisfaction (%)	61	80	0.08	63	71	0.56	63	50	0.23	65	83	0.16
Age of first sexual intercourse (yr)	22	21	0.35	22	21	0.15	22	20	0.35	21	20	0.26
Monthly sexual intercourse frequency	2.1	1.78	0.82	2.52	2.31	0.37	2.2	1.89	0.12	2.5	2.8	0.89

Impact of external genitalia appearance (typical female external genitalia at birth vs. atypical external genitalia) in 46, XY DSD females

The authors compared 95 females 46, XY DSD individuals divided according to the external genitalia appearance at birth. Sixty had typical female external genitalia (Sinnecker score 5), and 35 had atypical genitalia (Sinnecker score ranging from 2 to 4, median 3). There was no significant difference between groups in all sexual parameters (p > 0.05; Table 2). Then, the authors analyzed differences in the sexual parameters among female individuals with atypical genitalia (n = 35) at birth based on the Sinnecker score (II–IV), with no significant difference in any sexual life variable.

Male Gender in adulthood (46, XY individuals who were assigned as male vs. 46, XY individuals who changed from female to male social sex)

This comparison included individuals assigned as male (n = 20) and individuals who changed from female to male gender (n = 24) (Table 3). No significant difference in the sexual parameters was found between both groups, except for more active sexual life in adult males who changed from female to male than those assigned as male (75% vs. 96%; p = 0.04) (Table 2). Regarding sexual orientation, the frequency of non-heterosexuality was similar between both groups (Table 4).

**Table 3:** Clinical and molecular data of 46, XY DSD individuals who changed from female to the male gender.

Subject	Current age	Age of Gender- Change Desire (yr)	Age of gender change (yr)	Steady partner	Active sexual life	Gene	Zygosity	cDNA	Protein
1	26	6	15	Yes	Yes	SRD5A2	HH	c.547G>A	p.Gly183Ser
2	34	8	14	Yes	Yes	HSD17B3	HH	c.645A>T	p.Glu215Asp
3	51	5	22	No	Yes	HSD17B3	HH	c.645A>T	p.Glu215Asp
4	48	6	6	No	Yes	SRD5A2	HH	c.377A>G	p.Gln126Arg
5	32	8	8	Yes	Yes	SRD5A2	HH	c.337C>G	p.Leu113Val
6	29	7	15	Yes	Yes	SRD5A2	HH	c.547G>A	p.Gly183Ser
7	52	8	19	Yes	Yes	SRD5A2	HH	c.377A>G	p.Gln126Arg
8	55	6	6	No	Yes	SRD5A2	HH	c.763T>C	p.*255Glnext*28
9	40	8	15	Yes	Yes	SRD5A2	HT	c.763T>C/c.377A>G	p.*255Glnext*28/p.Gln126Arg
10	56	5	7	Yes	Yes	SRD5A2	HH	c.547G>A	p.Gly183Ser
11	25	5	7	Yes	Yes	SRD5A2	HH	c.547G>A	p.Gly183Ser
12	39	9	17	Yes	Yes	SRD5A2	HH	c.547G>A	p.Gly183Ser
13	60	10	31	No	Yes	SRD5A2	HH	c.679C>T	p.Arg227*
14	42	5	17	Yes	Yes	SRD5A2	HT	c.418del	p.Trp140Glyfs*20
15	43	5	13	No	Yes	SRD5A2	HT	c.200T>G/c.736C>T	p.I67S/p.Arg246Trp
16	42	5	14	No	Yes	SRD5A2	HH	c.547G>A	p.Gly183Ser
17	52	14	42	Yes	Yes	SRD5A2	HH	c.547G>A	p.Gly183Ser
18	24	2	5	No	Yes	SRD5A2	HT	c.200T>G/	p.I67S/p.R246W
19	50	9	27	Yes	Yes	HSD17B3	HT	c.608C>T/c.278-1G>C	p.Ala203Val / -
20	48	8	27	Yes	Yes	HSD17B3	HT	c.608C>T/c.278-1G>C	p.Ala203Val/c.278-1G>C
21	43	12	13	Yes	Yes	HSD17B3	HH	c.608C>T	p.Ala203Val
22	28	13	16	No	Yes	NR5A1	HH	c.1139-3C>A	‒
23	46	15	35	No	Yes	NR5A1	_	c.70C>T	p.His24Tyr / ‒
24	22	10	17	No	No	Unknown	Unknown	‒	‒

CA, Chronological Age; HH, Homozygosity; HT, Heterozygosity.

**Table 4:** Sexual orientation in 46, XY DSD individuals.

		Gender at adulthood			Female External Genitalia			Male Gender		
Sexual parameters		Female	Male	p	Typical	Atypical	p	Assigned as male	Changed to male	p
n		83	20		35	60		20	24	
Self-reported Sexual Orientation	Homosexual	4 (5%)	1 (5%)	0.34	1 (3%)	3 (5%)	0.28	1 (5%)	‒	0.33
	Bisexual	3 (4%)	1 (5%)		2 (6%)	1 (2%)		1 (5%)	1 (4%)	
	Heterosexual	76 (91%)	18 (90%)		32 (91%)	56 (93%)		18 (90%)	23 (96%)	
Sexual Behavior^a^	1	79	18	0.52	31	55	0.43	18	22	0.5
	2	‒	1		1	4		‒	1	
	3	‒	‒		‒	‒		‒	1	
	4	‒	‒		‒	‒		‒	‒	
	5	4	1		3	1		2	‒	

a To female gender individuals: 1 = exclusively women; 2 = more women than men; 3 = equal men and women; 4 = more men than women; 5 = exclusively men; and the opposite for male gender individuals: 1 = exclusively men to 5 = exclusively women). **NS, Non-Ssignificance.

### Impact of estimated prenatal androgen exposure on sexuality

Afraid of being romantically rejected was also more frequent among 46, XY DSD women with prenatal androgen exposure (p = 0.001), but there were no differences in the other sexual parameters (Table 2). However, the desire for motherhood in 46, XY DSD women with prenatal androgen exposure was significantly lower than those without this exposure (p = 0.032) – Table 5.

**Table 5:** Fertility issues according to prenatal androgen exposure and gender in adulthood.

Parameter	Prenatal androgen exposure in 46, XY Women			Gender in adulthood		
	Present	Absent	p	Female	Male	p
Motherhood/Fatherhood importance	1.72 ± 1.08	2.8 ±1.25	0.032	1.83 ± 1.02	2.26 ± 1.2	0.19
Infertility affects a steady relationship	3.21 ± 1.37	3.15 ± 1.05	0.32	3.51 ± 1.67	4.11 ± 1.2	**0.02**
Afraid of rejection due to infertility	21% (n = 9)	21% (n = 3)	0.81	32% (n = 25)	16% (n = 6)	**0.01**
The desire of biological children	79% (n = 32)	50% (n = 7)	0.04	79% (n = 60)	82% (n = 28)	0.25
Desire to adopt children	75% (n = 31)	36% (n = 5)	0.01	73% (n = 56)	75% (n = 24)	0.88

### Sexual orientation

According to self-reported sexual orientation, heterosexual orientation was referred by 91% (n = 76) and 90% (n = 18) of female and male individuals in adulthood, respectively (p = 0.18) (Table 4). Homosexuality was reported by 5% of the patients of both genders, whereas bisexuality by 4% in the male gender and 3% in the female gender (Table 4). Among individuals who changed from female to the male gender, 96% (n = 23) self-reported as heterosexual and 4% (n = 1) as bisexual. In the female gender, the frequency of heterosexuality was similar when considering the presence or not of atypical genitalia at birth (p = 0.17). In the male gender, the frequency of heterosexuality was similar between individuals assigned as male *versus* those who changed to the male gender.

### Gender change

Considering the sexual life of individuals who changed their gender, most (n = 23; 96%) reported an active sexual life and had experienced romantic relationships. Feelings of body shame were present in 37% (n = 9) of individuals who changed their gender, but it was not significant from those assigned as male (Table 2). The frequency of long-term romantic partners in these individuals was 62% (n = 15) compared to 40% of those assigned as male (Table 2). Twenty-five percent of assigned males reported feeling afraid of being romantically rejected, whereas only 8% of those who changed to the male gender reported this feeling (p = 0.07) (Table 2).

The authors obtained the molecular diagnosis in 23 out of 24 subjects who changed to the male gender (Table 3). The most frequent diagnosis was 5α-reductase type 2 deficiency (n = 16; 67%) followed by 17β-HSD3 deficiency (n = 5; 21%), partial gonadal dysgenesis (n = 2; 8%), and one patient remained with unknown etiology.

### Fertility issues

79% (n = 60) of women reported a desire to have children, whereas 82% (n = 28) of men reported this desire. A total of 12 individuals (7 males) had children. Among these, 10 (4 females and 6 males) out of 12 (83%) have adopted children. Two male individuals with 5α-reductase type 2 deficiency had their children after intravaginal insemination. Among individuals without children (n = 115; 90.5%), both women and men declared a desire to have children at a high frequency (79% and 82%, respectively).

Both women and men reported fear of being romantically rejected due to infertility, but more women than men reported this feeling (32% and 16%, respectively; p = 0.01). The importance of having children was greater among women than men (p = 0.01), and more women than men considered infertility a barrier to a long-term romantic relationship (p = 0.02).

## Discussion

This is the first Brazilian study evaluating the sexual aspects of a large cohort of DSD people diagnosed and followed in a single tertiary center. In this study, the mean chronological age of the onset of sexual life in both genders was later than the age reported in the Brazilian population.^13^ but similar to the previously described in 46, XY DSD individuals.^3^ The reasons for this delay are unclear, but body shame can be a barrier to the onset of sexual activity in 46, XY DSD subjects.^14^ About one-third of people with DSD in both genders reported feeling ashamed of their own bodies[3] and 25% of DSD people attributed their difficulty in being engaged in a romantic relationship primarily to their physical appearance.^15^ Therefore, the authors speculated that DSD people might spend more time choosing a romantic partner with whom they feel safe to start a sexual relationship, which could justify the late beginning of their sexual life.

As far as sexual life satisfaction is concerned, individuals with 46, XY DSD reported less sexual satisfaction than controls in the literature,^3^^,^^16^^,^^17^ regardless of gender. Indeed, several psychological issues related to the DSD condition can negatively impact the sexual quality of life, such as social stigmatization, reduced self-esteem, infertility, and previous traumatic experiences of DSD individuals.^18^^,^^19^ The authors identified that sexual fantasies and masturbation practices were more frequent among 46, XY DSD males than females. Our data agree with other studies on masturbation, reporting consistent data that non-DSD men practiced more masturbation than women,^20^^,^^21^ suggesting that the same happened among DSD people.

While sexual life satisfaction has been evaluated in several DSD studies, the results are not consistent, by differences in the number of patients enrolled, the profile of 46, XY DSD etiologies, and the methodologies applied.^3^^,^^22^^,^^23^ Among 46, XY DSD people, the frequency of having a long-term loving partner is quite variable. Studies show a similar frequency of long-term romantic partners among people with and without DSD ^19^^,^^24^^,^^25^ and other studies showed a lower frequency of long-term partners among DSD than non-DSD people.^3^^,^^5^^,^^19^ Our data demonstrated a high frequency of sexual partners in both genders. In the literature, there are pieces of evidence showing that males 46, XY DSD male had a long-term romantic partner more frequently than females 46, XY DSD.^3^^,^^5^^,^^16^ Among females, it is noteworthy that the frequency of a long-term loving partner was higher among those without prenatal androgen exposure (such as complete androgen insensitivity and complete gonadal dysgenesis) than those with this exposure.

In this cohort, most individuals from both genders referred to having previous romantic partners showing that 46, XY DSD people engaged in loving relationships. At the time of this investigation, there were fewer 46, XY DSD women than 46, XY DSD men in a current romantic relationship, but most of them referred to an active sexual life. Among 46, XY DSD women, 70% referred to having active sexual life, contrasting with the low frequency of women with a long-term romantic partner (40%). Those results indicate that 46, XY DSD women dissociated from active sexual life and long-term romantic relationships.

It has been reported that the sexual life of 46, XY DSD women with typically female genitalia at birth is better than patients with atypical genitalia.^5^^,^^26^ Nonetheless, the authors did not find any difference in sexual aspects comparing 46, XY DSD women presenting atypical or typical female genitalia at birth. It suggests that external genitalia appearance did not interfere after feminizing surgery in their sexual life and sexual activity.^27^^,^^28^

Here, gender change from female to male occurred in 22% (24 out 107) of the patients. The most frequent 46, XY DSD diagnosis related to gender change was 5α-RD2 deficiency. Indeed, individuals with 5α-RD2 deficiency were commonly assigned as female due to the severe undervirilization of the external genitalia.^9^^,^^29^ It is important to note that the gonads can produce testosterone in this condition, which does not need to be converted to DHT for brain virilization.^9^^,^^30^^,^^31^ Brain virilization occurs predominantly during prenatal time and does not happen simultaneously with external genitalia virilization.^32^^,^^33^ Therefore, male gender identity is common among them, regardless of the female-like external genitalia appearance. Consequently, gender change from female to male is common in this 46, XY DSD condition.^31^ Gender change from female to male is also reported among other 46, XY DSD conditions but in a lower frequency.^6^^,^^8^^,^^34^^,^^35^ In our analysis, there were no differences in the sexual life parameters comparing male individuals assigned as male with those who changed from female to male, except individuals who changed from female to male gender reported a more active sex life than those assigned as male (p > 0.04; Table 2). In the literature, data from transgender men (non-DSD) report a high frequency of active sexual life after gender-affirming surgery.^36^ However, this difference might be explained by the 46, XY DSD diagnosis. Most of those who changed from female to male had 5α-RD2 deficiency, whereas most were assigned as male had PAIS (Table 1). There are few results in the literature exploiting the sexual life of male 46, XY DSD individuals based on DSD etiology, but these results have suggested that each 46, XY DSD etiology can impact male sexuality differently. While individuals with 5α-RD2 deficiency reported satisfaction with their sexual activity,^37^ regardless of the penile length,^4^ 86% of men with partial androgen insensitivity had sexual life dissatisfaction.^38^ The reason is still unclear, but it is speculated that gynecomastia and the absence of complete body virilization affect their sense of masculinity with consequences on their sex life.^6^

Despite no significance (p = 0.07), it is worth noting that individuals assigned as the male had more afraid of being romantically rejected (25%) than those who changed from female to male gender (8%; Table 2).

The effects of prenatal androgen exposure on human psychosexuality have been reported in the literature.^7^^,^^39^, ^40^, ^41^, ^42^, ^43^ Our data showed that individuals with prenatal androgen exposure were more afraid of being romantically rejected than individuals without androgen exposure. Interestingly, the frequency of long-term romantic partners was similar between both groups, suggesting that females with prenatal androgen exposure engaged in a long-term romantic relationship as frequently as those without this exposure, regardless of their fear of rejection.

Infertility is another relevant issue in managing DSD people.^44^, ^45^, ^46^ In the past, infertility was a rule for most of the 46, XY DSD conditions, impacting their self-acceptance and well-being.^47^ Fortunately, assisted reproductive techniques have opened up possibilities of parenthood for DSD subjects.^44^^,^^45^ In our cohort, most individuals from both male and female gender had fertility desire, and almost all of them considered adopting a child to fill their desire for parenthood. However, more 46, XY DSD women than 46, XY DSD men were afraid of being romantically rejected due to infertility, and more women than men attributed to infertility their difficulties in establishing a long-term romantic relationship (Table 5). It might explain the lower frequency of 46, XY DSD women within a long-term romantic relationship in this cohort in comparison. With 46, XY DSD men (Table 2). The importance of motherhood was lower among females with prenatal androgen exposure than 46, XY DSD women without prenatal androgen exposure, suggesting that this exposure plays a role in females' motherhood desire. Pieces of evidence of this association came from studies about congenital adrenal hyperplasia (CAH). CAH due to 21 hydroxylase deficiency affecting 46, XX newborns has been successfully studied as a model to prove the role of androgens in many human sexual characteristics and behavior.^40^^,^^48^ It has been demonstrated that CAH diagnosis impacts on parenthood desire of affected individuals,^49^ reinforcing the negative association between prenatal androgen exposure and parenthood desire. Our results showed that prenatal androgen exposure also impacts motherhood desire in 46, XY DSD women.

To explore infertility issues in DSD, a large cross-sectional multicenter study applied a web questionnaire in DSD people (46, XX and 46, XY DSD).^46^ In this study, 14% reported having at least one child including 7% with assisted reproductive techniques and 4% adopted. Our cohort found that only 9% (n = 12) of 46, XY DSD people had children. In most cases (10 out of 12), those children were adopted. Curiously, there is a discrepancy in the percentage of 46, XY DSD individuals who desire children and the frequency of those who had adopted children. Indeed, the process of adopting a child is often bureaucratic in many countries, including Brazil, which may partially explain this discrepancy. However, future fertility expectancy was one reason why 46, XY DSD women due to complete androgen insensitivity deferred gonadectomy.^50^ Therefore, discussion on fertility desire and current options for fertility is strongly recommended for all DSD individuals to promote appropriate perspectives, avoiding further frustrations and negative impact on their quality of life.

Human sexual orientation is defined based on self-identification (heterosexual, homosexual), sexual attraction (the gender in which sexual desire is driven), and sexual behavior (based on the gender of the sexual partners).^51^ Regarding sexual attraction, the literature shows that most 46, XY DSD men are gynophilic (erotically attracted to women), whereas most 46, XY women are androphilic (erotically attracted to men).^52^^,^^53^ Self-reported sexual orientation is the most usual way used to assess sexual orientation. However, patients usually inform their sexual orientation based on sexual behavior (gender of their sexual partners) instead of considering their erotic attraction and fantasies.^54^^,^^55^ Available data on sexual orientation in 46, XY DSD people have shown more frequency of heterosexuality in both genders,^3^^,^^15^^,^^17^^,^^22^ but the frequency of non-heterosexuality is higher than matched controls, especially in conditions with prenatal androgen exposure.^7^^,^^48^^,^^56^

The external genitalia appearance and the related stigma are relevant for many DSD patients.^57^ Genital ambiguity may cause difficulties with sexual intercourse, social stigma, reduced self-esteem, and avoidance of nudity, dating, and sexual relations.^22^ In this study, the authors included 46, XY women assigned female sales at birth with or without atypical genitalia at birth. Among those with atypical genitalia at birth, all of them have completed all genital feminizing surgical procedures at the time of this study. The presence of atypical genitalia at birth in 46, XY DSD women did not increase the frequency of non-heterosexuality, and the sexual parameters (including masturbation practice, orgasm, and active sexual life) were similar among these women. While the timing of feminizing genital surgery in DSD remains debatable, data on the sexuality of female DSD patients is scarce. Our results suggest that sexual life is similar among 46, XY DSD women regardless of their genitalia appearance at birth, once all feminizing genitalia surgeries have been performed.

## Limitations

The lack of a control group composed of non-DSD 46, XY people is a limitation.

However, our results allowed us to estimate differences and similarities among 46, XY DSD subgroups and the influence of variables (such as external genitalia appearance, prenatal androgen exposure, and gender change) in the sexuality of 46, XY DSD people. As 46, XY DSD is a rare and very heterogeneous condition, lack of difference might result from a lack of statistical power to detect changes. Therefore, the authors cannot rule out a type 2 statistical error. In addition, the particularities of each 46, XY DSD condition on sexuality were not evaluated in this study, which should be explored in future research.

In conclusion, the desire for fertility is shared among 46, XY DSD people, regardless of gender. Therefore, it should be taken into account in the 46, XY DSD management. Prenatal androgen exposure reduces the desire for motherhood in 46, XY women. Among males, individuals who changed to the male gender presented similar sexual parameters as those assigned as males. Among females, virilized genitalia at birth did not affect their sexuality once the surgical treatment is completed. Collectively, these results can expand the knowledge about sexuality in 46, XY DSD people.

## Ethical approval

This study was approved by the local Ethical Committee (Hospital das Clínicas da Universidade de São Paulo).

## Conflicts of interest

The authors declare no conflicts of interest.
